# Digital Literacy Training for Low-Income Older Adults Through Undergraduate Community-Engaged Learning: Single-Group Pretest-Posttest Study

**DOI:** 10.2196/51675

**Published:** 2024-05-14

**Authors:** Lisa M Soederberg Miller, Rachel A Callegari, Theresa Abah, Helen Fann

**Affiliations:** 1 Human Ecology University of California, Davis Davis, CA United States; 2 Department of Gerontology Sacramento State University Sacramento, CA United States

**Keywords:** community-engaged learning, digital divide, underserved older adults, digital literacy training, intergenerational programs

## Abstract

**Background:**

Digital technology is a social determinant of health that affects older people’s ability to engage in health maintenance and disease prevention activities; connect with family and friends; and, more generally, age in place. Unfortunately, disparities in technology adoption and use exist among older adults compared with other age groups and are even greater among low-income older adults.

**Objective:**

In this study, we described the development and implementation of a digital literacy training program designed with the dual goals of training low-income older adults in the community and teaching students about aging using a community-engaged learning (CEL) approach.

**Methods:**

The training program was embedded within a 10-week CEL course that paired undergraduates (N=27) with low-income older adults (n=18) for 8 weeks of digital literacy training. Older adults and students met weekly at the local senior center for the training. Students also met in the classroom weekly to learn about aging and how to use design thinking to train their older adult trainees. Both older adults and students completed pre- and posttraining surveys.

**Results:**

Older adults demonstrated increased digital literacy skills and confidence in the use of digital technology. Loneliness did not change from pre to postassessment measurements; however, older adults showed improvements in their attitudes toward their own aging and expressed enthusiasm for the training program. Although students’ fear of older adults did not change, their comfort in working with older adults increased. Importantly, older adults and students expressed positive feelings about the trainee-trainer relationship that they formed during the training program.

**Conclusions:**

A CEL approach that brings together students and low-income older adults in the community has a strong potential to reduce the digital divide experienced by underserved older adults. Additional work is needed to explore the efficacy and scalability of this approach in terms of older adults’ digital literacy as well as other potential benefits to both older and younger adults.

## Introduction

### Background

Digital technology is a social determinant of health that plays a significant role in older adults’ lives, including their ability to engage in health maintenance and prevention activities; connect with family and friends; and, more generally, age in place [1-3]. Recent evidence from the COVID-19 pandemic indicates that the digital divide contributed to health inequities among older individuals who were unable to benefit from technology to support health and well-being when in-person alternatives were unavailable or unsafe [4-7]. For example, without adequate online alternatives, older adults experienced greater health challenges and increased social isolation [8,9]. Conversely, older adults who had technology support during the pandemic fared better. For example, results from a qualitative study within a continuing care community indicated that technology mitigated social isolation and loneliness during the pandemic [10].

Disparities in technology adoption and use are particularly pronounced among older adults compared with other age groups [11-13] and are even greater among low-income older adults [3,14]. Some evidence suggests that internet use among low-income older adults may be as low as 17% [11] and that health-related technology use is significantly lower among racial and ethnic minority older adults as well as among low-income older adults [15]. Barriers that contribute to low rates of technology use among older adults include broadband availability, cost of broadband and devices, lack of awareness of potential technology benefits, low self-efficacy, and lack of training [16,17]. Some estimates indicate that only 25% of older residents in low-income housing have reliable internet access [18]. However, another study on low-income housing residents showed that although the housing communities in the study had access to broadband, few of the residents used the internet [19]. Thus, providing broadband and a digital device is insufficient. Digital literacy training is particularly important within this segment of the population to overcome barriers and promote sustained engagement [20].

### Digital Literacy Training

There have been numerous approaches used to teach older adults how to use technology [16,21-23]. Regardless of the approach, researchers tend to agree that hands-on training over multiple weeks and tailored training programs are particularly important to meet the needs of older adults [24]. Tailoring can be achieved in a variety of ways—either informally (eg, drop-in or as-needed help) or as part of an in-home or a classroom-style program in the community [25-29].

Two theoretical models of technology acceptance and use among older adults are particularly well-suited to promoting digital literacy in this population [16,24,30]. First, the Senior Technology Acceptance and Adoption Model (STAM) includes ease-of-learning and ease-of-using technology constructs and argues that these are significant drivers of actual use [31]. Second, the Center for Research and Education on Aging and Technology Enhancement (CREATE) model of technology use in later life focuses on older adults’ characteristics, including demographics and psychographics (psychosocial background), cognitive, perceptual, and psychomotor abilities. The model also considers the relationship between older adults’ capabilities and the demands of the technology task being performed (eg, more challenging tasks undertaken will require greater capabilities) [16,32]. Thus, the CREATE model informs training by highlighting the importance of the fit between trainee and training activities as well as the rate at which training proceeds.

**Figure 1:**
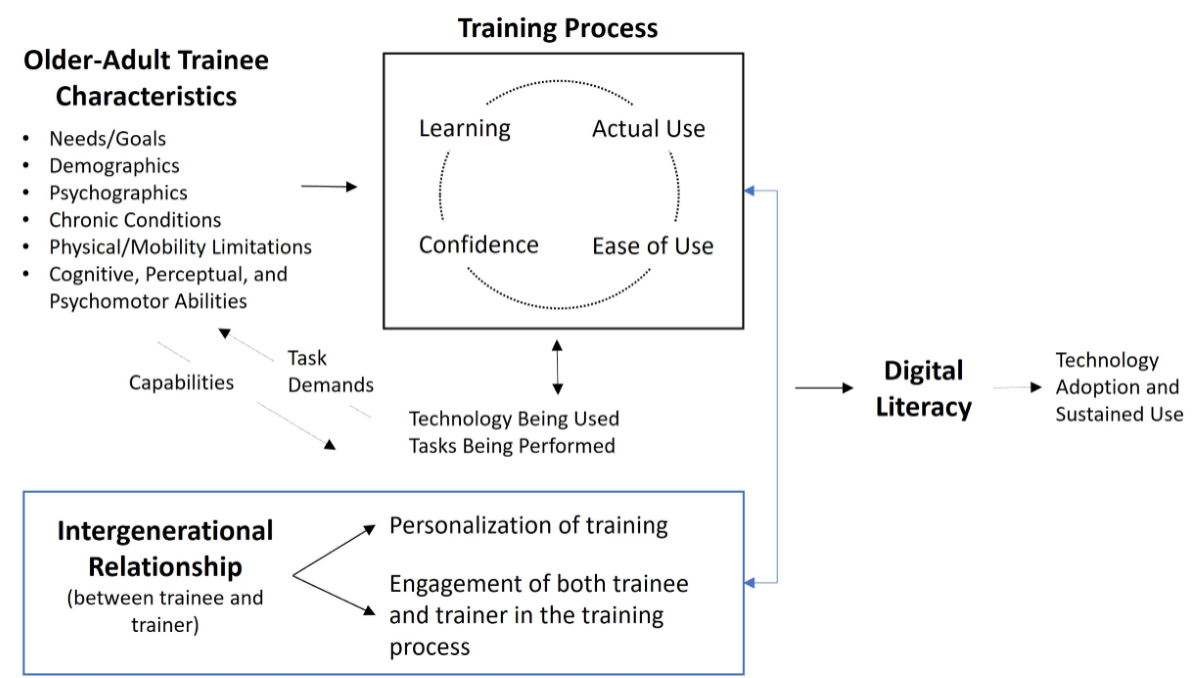
Conceptual model of digital literacy training.

Drawing on these 2 models, we developed a conceptual model of digital literacy training, which is shown below in Figure 1. First, our conceptual model incorporates the STAM’s [31] close connection between ease of use on the one hand and actual use on the other. Last, the conceptual model places training at the center of adoption as being critical for low-income older adults who are far less likely to have prior experience with technology use and learning to use technology. The model provides a starting place for considering how training can be tailored to meet the needs of older adults with varying backgrounds and capabilities. Second, our model relies heavily on the CREATE model’s [32] emphasis on older adults’ characteristics (eg, demographics and cognitive and perceptual abilities) and the importance of the relationship between older adults’ capabilities and the demands of the task being performed.

Training programs targeting low-income older adults are far less common in the literature. Nevertheless, research suggests that factors that support older adults in general may be even more important for older adults from low-income backgrounds who may be reticent to seek training. Given the wide-ranging prior experience with technology and learning environments, a higher degree of tailoring and personalization may be critical to training those with lower-income and education backgrounds [20,24,33-35]. In addition to the insecurities surrounding the use of technology, low-income older adults may be more likely to have insecurities surrounding their ability to learn to use a computer, which in turn can impact enrollment into educational programs [33]. Low-income older adults may also lack awareness of the potential benefits of technology, which could also negatively impact motivation to learn to use technology and to persist in learning, especially when challenges arise [36,37]. For these reasons, programs that provide one-on-one training with highly individualized content and pacing may be a particularly important way to engage this population of older adults in training programs. Textbox 1 shows the training features, supported by the literature reviewed above, which may be particularly well-suited to low-income older adult populations.

**Evidence-based digital literacy training features proposed for low-income older adults.**

**Digital literacy training program features**

- Hands-on (trainees use their device while being instructed)
- Tailored to the specific needs and goals of the trainee
- Personalized to optimize trainee engagement
- Delivered one-on-one (supports demonstration, practice, immediate feedback, and question and answer [Q&A] time)
- Trainee completes practices exercises in between meeting times; experience (successes and failures) is reviewed at subsequent meetings
- Same trainee-trainer pairs work together across training programs to foster trust and respect through relationship building

### Intergenerational Approach to Digital Literacy Training

Another approach with the potential to engage and motivate older adults in digital literacy training, particularly older adults from underserved communities, is to provide opportunities for intergenerational relationships within the training process [38,39]. Intergenerational programs involve a younger generation (eg, college students) interacting with older adults who are typically from the same community. Opportunities in which different cohorts or age groups interact are becoming increasingly less frequent in society due to age segregation and changing family structures. In an age-segregated society, education, work, and leisure are apportioned to younger, middle, and older ages, respectively, restricting opportunities for older adults to spend time with younger individuals [40]. Intergenerational opportunities are further limited by changes in family structure, including fewer children and an increased likelihood of relocating to pursue a job or other opportunities [41]. Intergenerational programs often focus on fostering cooperation, interaction, and exchange between generations and can provide benefits, such as improved social connectedness and attitudes toward aging for both students and older adults [42-47].

Support for an intergenerational approach to digital literacy training comes from a study in which 38 pairs of high-school students and older adults met 2 or 3 times for 1.5 hours of training [21]. Although the study did not include digital literacy outcomes, results showed satisfaction for both the students and older adults. In another study, researchers explored 3 approaches to digital literacy training with students (undergraduate upperclassmen students in professional programs [eg, pharmacy]) and older adults [48]. With the first approach, students trained older adults in a 30- to 60-minute appointment at the senior center on an as-needed basis. The second approach matched students in a gerontology class with older adults from a local Osher Lifelong Learning Institute for a minimum of 6 hours of training at times and places determined by each trainer-trainee pair. The third approach consisted of as-needed, drop-in sessions held by students in 2- to 4-hour time blocks at the Osher Lifelong Learning Institute. Results showed that among older adult trainees who attended ≥3 sessions, trainees valued the intergenerational relationships developed over the training period as well as increased interest in working with technology. Moreover, younger adults showed improvement in attitudes toward aging, confidence in teaching older adults, and comfort working with older adults [48]. Similar to the matching program in Leedahl et al [48], Arthanat [49] paired undergraduate older adults in an occupational therapy degree program with older adults for 3 months of training (8 training sessions total) as part of a service learning project for an assistive technology course. Again, the training schedule was set by each pair. Before training, students attended a laboratory session on technology and aging and were encouraged to use Facebook as a forum to connect with other student trainers across the semester. Researchers reported increases in older adults’ frequency of technology use for multiple purposes, including those related to health and hobbies, and self-reported independence in a range of digital activities, reflecting improved digital skills. In general, the data show that intergenerational programs can effectively engage older adults in digital literacy training.

Programs that bring together students and members of the community provide a critical opportunity for community-engaged learning (CEL). CEL incorporates activities typically associated with internships and service learning into formalized learning within courses that consider social needs and social changes in the community. It also emphasizes the significance of building relationships with individuals in the community to bring about social change [50]. Young adults are often at the forefront of social change, with a heightened interest in the broader world, where they see themselves in society and how they can make a difference [51]. Intergenerational CEL provides an opportunity for younger individuals to broaden their awareness of social needs by exposing them to issues related to digital exclusion among underserved older adults. Textbox 2 describes the intergenerational training features supported by the literature.

**Summary of intergenerational digital literacy training used in this study.**

**Intergenerational content and features**

- A community-engaged learning framework was used that emphasizes providing students with the opportunities to develop academic skills, civic competencies, and ethical commitments while exploring community-based efforts to address social justice issues.
- The training program was integrated into a structured course with set weekly meeting times for (1) student classroom learning and (2) training older adults; set times remove weekly scheduling burden and uncertainty regarding the training schedule.
- Same trainer-trainee pairs worked together across the training program to enable the trainer to get to know the trainee to foster a trusting relationship that is conducive to frank and open discussion about training needs and pace.
- Weekly student classroom time used to teach students about aging (agism; technology as a structural determinant of health with cumulative disadvantage perspective; age-related changes in perceptual, cognitive, and motoric/physiological capabilities) and other factors that may impact older adults’ acceptance, adoption, and sustained use of technology.
- Student classroom time was also used to teach students about design thinking and how to apply design thinking to designing personalized and tailored training for their older adult trainee.

### Background Work

Before this study commenced, we explored the logistics of pairing students and older adults in the community within an existing course on aging and technology use. Students (n=30) were partnered with older adults (n=17) in small groups (typically, 3 students and 1 older adult) over 4 to 5 weeks. Students were responsible for contacting older adults by telephone (using a free app such as Google Meet) and scheduling weekly 30- to 45-minute meetings. Telephone discussions focused on older adults’ current use of technology, attitudes toward technology, and what their preferences for training would be if they were to seek training. Students made notes each week to track what they learned about their partner’s technology acceptance. At the end of the quarter, we asked both students and older adults to answer open-ended questions about the challenges and rewards of their student-partner interactions. Overall, we learned that both the students and older adults highly valued their time together, and their relationships grew as they got to know each other. However, scheduling weekly appointments took a substantial amount of time, due to difficulties in identifying time slots, cancelations, and rescheduling.

### This Study

This study examined a digital literacy training program integrated within an intergenerational CEL course to explore a formalized pathway connecting undergraduate education and underserved older adults in the community. The digital literacy training program was embedded within a formalized course structure in which students received college credits as they learned about aging and social justice while working one-on-one with low-income older adults in the community. The training program, designed to work within a course structure rather than in parallel or as an add-on (eg, with a minimal connection between class time and training time), offered 3 additional benefits for student trainers and older adult trainees. First, a set time and place removed uncertainty in scheduling from week to week. Second, weekly contact between students and the instructor in the classroom provided time to teach students about aging, social justice, and digital literacy training. Third, weekly contact between students and older adults at the training location (ie, local senior center) allowed for meaningful relationship building. Fourth, the presence of the instructor at the training site supported the students and older adults by facilitating communication and troubleshooting should any problems arise. Overall, this provided students and older adults with formalized and consistent support and, potentially, higher-quality education for the students and training for older adults. Finally, we offered the CEL course as a first-year seminar (with no prerequisites) in an attempt to attract students early in their academic studies. Students from any majors (including “undeclared” majors) were able to review the list of first-year seminars and sign up for those they wished to take.

In pairing students with low-income older adults in the community over an 8-week period, we hypothesized that relationships would develop organically as the intergenerational pairs worked together. The CEL course included features to support the process and the course used “design thinking” to encourage students to consider the older adult holistically on how technology fits within this individual’s life. Design thinking is a human-centered approach that places the “user” at the core when solving “wicked” or ill-defined problems, such as how to design a digital literacy training program that is well-suited to the trainee [52,53]. The course also taught students about aging (eg, older adult characteristics shown in Figure 1), stereotypes and biases related to aging, and how to train older adults using a design thinking approach, which focuses on understanding the end user and defining the task at hand (ie, designing an effective training program for their trainee).

## Methods

### Participants

#### Undergraduate Students

There were no prerequisites and no restrictions on who could sign up for the course. Among the undergraduate students (N=26) in the course, 48% (n=13) were female individuals, and 77% (n=21) were underclassmen (ie, freshman or sophomore), and they represented a wide range of majors, including data science, mathematics, sociology, and animal biology.

#### Older Adults

Older adults (N=23) were recruited with the support of a local nonprofit organization as well as low-income housing organizations and the local senior center. Inclusion criteria consisted of older adults who were aged ≥60 years; were eligible for a federal or state safety-net program (eg, Meals on Wheels, senior low-income housing, Medi-Cal, Cal-Fresh [SNAP]); were residents in Yolo County; and had little-to-no prior experience with computers. Funds provided by the County helped pay for recruitment and enrollment support as well as new laptop computers (which the participants were allowed to keep) together with 2-year internet subscriptions for the low-income older adults in the training program. Screening resulted in 5 of 23 individuals being excluded (1 for not meeting the age criterion and 4 for not meeting the low-income criterion). The final sample of older adults (n=18) was predominantly female (n=17, 95%) and non-Hispanic White (n=10, 55%), with the remainder being Asian (n=4, 22%) and Hispanic White participants (n=4, 22%). Older adults’ age ranged from 61 to 87 (mean 72, SD 7.81) years and had a mean of 17.61 (SD 5.21) on the Lubben Social Network Scale, which assesses social networks for family and friends with possible a range of 6 to 36 and clinical cutoff of ≤12 [54].

### Ethical Considerations

The study was deemed exempt by UC Davis’s institutional review board. Older adults were read an information script before the pretest, informing that they could quit anytime and that their individual-level data would be deidentified.

### Measures

#### Undergraduate Student Pretest-Posttest Measures

##### Psychological Growth

The Psychological Growth scale (8 items) from the Attitudes to Aging Questionnaire [55] was used to assess students’ attitudes toward aging. The scale includes items, such as, “It is a privilege to grow old,” “As people get older they are better able to cope with life,” and “There are many pleasant things about growing older.” Responses are made on a 5-point Likert scale (1=strongly disagree; 5=strongly agree) and are summed, with higher scores reflecting more positive attitudes toward aging.

##### Fear of Old People

The Fear of Old People subscale of the Anxiety about Aging Scale [56] was used to assess students’ attitudes surrounding intergenerational relations. The subscale includes 5 items, such as “I enjoy being around old people” and “I like to go visit my older relatives,” with a 5-point Likert scale (1=strongly agree; 5=strongly disagree). Items were summed, with higher scores indicating more anxiety toward aging.

##### Working With Older Adults

We included 3 items to assess students’ attitudes toward working with older adults as they trained older adults [48]. The items were “I am comfortable working with older adults,” “I am confident in teaching older adults how to use technology,” and “I am likely to volunteer in the field of senior services,” with responses on a 5-point Likert scale (1=strongly disagree; 5=strongly agree).

#### Undergraduate Student Posttest-Only Measures

##### Rank Order

Students rank-ordered five aspects of the course from most important to least important: (1) learning about aging; (2) human-centered design and design thinking; (3) community engagement; (4) working with older adults; and (5) getting to know their trainee, specifically.

##### CEL Values

CEL addresses a specific community interest, problem, or public concern; includes working with and learning from a community partner; connects and integrates community-engaged experiences with educational content; and includes structured critical reflection. Students were given the following prompt: “Now that you’ve experienced CEL, we would like your perspective on its value. Please indicate how important the following CEL characteristics are to you,” followed by 12 items, such as “It makes me a better student in the long run” and “I want to contribute to the good of our society,” with responses on a scale of 1 to 3 (not very important to me, score=1; neutral, score=2; and very important to me, score=3).

#### Older Adult Pretest-Posttest Measures

##### Technology Skills

The Mobile Device Proficiency Questionnaire (MDPQ-16) was adapted to focus on “laptop” proficiency rather than mobile devices [57]. The questionnaire includes 8 scales, each assessing how easily the individual can perform digital tasks (n=18), such as navigating onscreen menus using the touchscreen, sending pictures by email, finding health information on the internet, and entering events and appointments into a calendar with responses of never tried (score=1), not at all easily (score=2), not very easily (score=3), somewhat easily (score=4), and very easily (score=5).

##### Loneliness

Loneliness was assessed using the 3-item Loneliness Scale [58], which asks how often individuals feel they lack companionship, are left out, and are isolated from others, on a scale ranging from hardly ever (score=1) to often (score=3). Scores are summed to create an overall assessment of loneliness. People who score 3 to 5 are considered “not lonely,” whereas people who score 6 to 9 are considered “lonely.”

##### Attitudes Toward Own Aging

Attitudes Toward Own Aging is a subscale of the Philadelphia Geriatric Center Morale Scale [59]. Respondents are asked to indicate whether they agree (score=1) or disagree (score=0), with 5 statements about aging such as “Things keep getting worse as I get older” (reverse scored) and “I have as much pep as I had last year.”

#### Older Adults Posttest-Only Measures

##### Relationship Quality

On a scale of 1 to 5 (1=strongly disagree; 5=strongly agree), older adults were asked to rate the following: “To what extent do you agree with the following statements about working with the students?”; “I enjoyed working with the student(s)”; “I feel that I formed a close relationship with the student(s)”; and “I will miss interacting with the student(s) now that the project has ended.”

##### Social Connectedness During Training

Older adults were asked how they feel about social connectedness using the following statement: “Did you feel more socially connected during the technology training program?” Responses were rated on a 3-point scale (1=no, 2=a little, and 3=a lot).

##### Self-Efficacy and Confidence

Three items on the extent to which participants agree with statements on computer skill improvement and confidence were as follows: “Your laptop skills have improved.”; “You are more independent when using your laptop.”; and “Your confidence in using laptop has improved.” Responses were rated on a 5-point scale (1=strongly disagree to 5=strongly agree).

##### Overall Evaluation

For overall evaluation, older adults were asked the following questions: “To what extent do you feel the training program was a positive experience” and “To what extent do you feel the training program was a valuable experience” with responses rated as 1=not positive or valuable to 5=very positive or valuable; “How likely would you be to recommend this technology training program to others?” with responses rated as 1=extremely unlikely to 7=extremely likely.

### Procedure

#### Course

**Figure 2:**
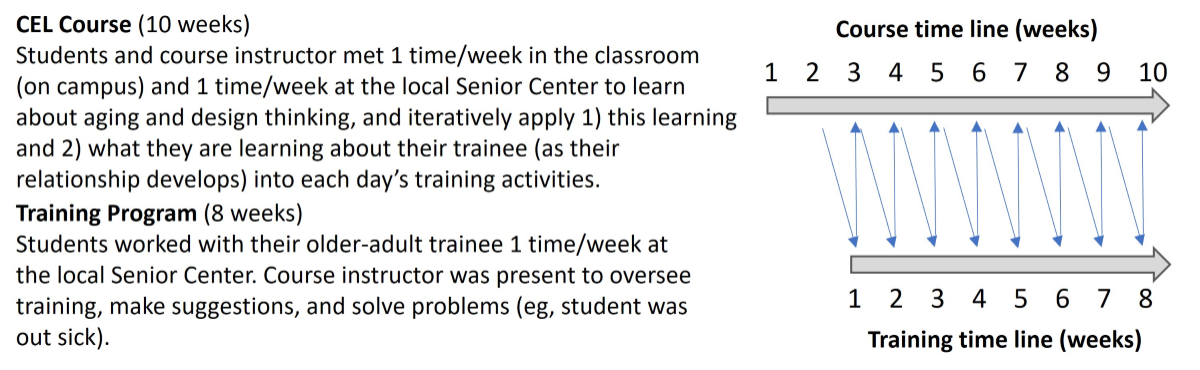
Illustration of how the digital literacy training was integrated into the community-engaged learning (CEL) course.

Undergraduate students enrolled in the CEL course in September 2022. In the first 2 weeks of the course, students (in groups of 1 or 2) were paired with an older adult so that each intergenerational group could begin to work together starting in week 3 of the course (which was week 1 of the training program) and continue to work together for the entire 8-week training program. Students met in the classroom on Tuesdays (50-min class) and in the senior center on Thursdays (50-min session), where older adults joined them for training. Figure 2 illustrates the back-and-forth nature of the education (CEL course) and training program and the overlap between the ten 50-minute classroom meetings and eight 50-minute training sessions at a nearby senior center where the training was conducted.

Each week, students submitted 2 assignments. First, they submitted their reflections on how the training process is going, observations about needs for subsequent training, and what they learned about their trainee as a person. The last part (what they learned about their trainee) formed the basis for our design thinking approach, which placed heavy emphasis on understanding older adults, including where and with whom they live, family members, motivations and needs for technology, and any other information that enabled the student to understand (get to know) the older adult trainee. Second, students submitted a training record that documented each task and their step-by-step instructions. The training records were combined to create a learner booklet for the trainee at the end of the training program.

Students completed the pretest during the second week of the quarter and the posttest during the week 9 of the quarter via Qualtrics (Qualtrics International Inc) online software. For older adults, a research assistant administered the pretest via telephone during weeks 1 and 2 of the training program and the posttest 1 to 2 weeks following the training.

#### Digital Literacy Training

A nonprofit community partner, Yolo Healthy Aging Alliance, provided trainees with (1) funds to purchase broadband internet for 2 years and (2) a Chromebook (a relatively inexpensive laptop costing about US $150 each, with a larger keyboard and screen than most tablets), which trainees used during training and were allowed to keep after the training. At the first session, older adults were introduced to their student trainers and were given their new laptops. Students then helped the older adults establish a user account and practice using the keyboard and touchpad to navigate to various places (note: due to an error, computer mice were not delivered until after the third week). Starting with a list of tasks that were commonly mentioned in the preliminary study and in the literature (eg, how to send and receive emails), students worked with their older adult trainees in the subsequent sessions to determine an appropriate pace and topics of importance to the trainee. See Multimedia Appendix 1 for the overview provided to students. After each session, students created step-by-step instructions for each task that they covered that day, including reminders and screenshots with arrows to direct attention. Students shared these instructions with trainees at the following session to obtain their feedback on clarity and granularity (level of detail) and adjusted the subsequent instructions as needed to maximize the older adults’ learning. At the end of the training program, students presented these learner booklets to their trainees as a PDF document in hard copy and digital form (via email).

## Results

### Undergraduate Students

#### Pretest-Posttest Analyses

Table 1 shows the means for all the pretest-posttest measures. Students’ scores on the Psychological Growth scale of the Attitude on Aging Questionnaire were summed to create a summary score for each time point. We found that scores did not significantly change from the beginning (mean 29.58, SD 3.11) to the end of the course (mean 29.19, SD 3.86; t_25_<1, *P*=.46). Similarly, the sum of items on the Fear of Old People scale did not change significantly from the beginning (mean 10.19, SD 2.70) to the end of the course (mean 9.81, SD 2.95; t_25_<1, *P*=.42). Student ratings of comfort working with older adults significantly improved from pretest (mean 4.00, SD 0.57) to posttest measurements (mean 4.27, SD 0.45; t_25_=−3.04, *P*=.006). Neither confidence in teaching older adults how to use technology (t_25_=1.31, *P*=.20) nor likelihood of volunteering in the field of senior services (t_25_=1.22, *P*=.23) significantly changed.

**Table 1:** Pretest-posttest summary variables for students (N=26).

Pretest-posttest variables (range of possible scores)	Pretest measurements, mean (SD)	Posttest measurements, mean (SD)	*t* test (*df*=25)	*P* value
Psychological growth (8-40)	29.58 (3.11)	29.19 (3.86)	0.73	.46
Fear of old people (5-25)	10.19 (2.70)	9.81 (2.95)	0.82	.42
Comfort working with older adults (1-5)	4.00 (0.57)	4.57 (0.54)	−3.04	.006
Confidence in teaching older adults (1-5)	4.15 (0.54)	3.96 (0.72)	1.31	.20
Likelihood of volunteering in the field of senior services (1-5)	3.38 (0.98)	3.58 (0.95)	−1.22	.23

#### Posttest Analyses

The end-of-course rankings of course features showed that students preferred working with older adults in the community over other course features: working with their older adult trainees was ranked first by 46% (12/26) of students and ranked first or second by 70% (18/26) of students. Working with older adults in general or specifically with their trainees was ranked first by 77% (20/26) of students. Learning about aging ranked the lowest with 77% (20/26) of students placing it in the bottom 2 positions. CEL rankings dropped in the middle (12/26, 46%, in the third position), and human-centered design rankings were evenly distributed across the 5 positions. Last, endorsements of CEL statements showed that students particularly valued contributing to the good of society, with 88% (23/26) indicating that this is very important to them (highest endorsement). The following 3 statements received *very important* ratings from 84% (22/26) of the students: “It helps me build compassion for myself and other people,” “The skills and knowledge that I gain will help me in my career,” and “I build relationships with people who live and think differently than I do.” In total, 80% (21/26) rated “It makes me a better student in the long run” as being very important. The following 2 statements received the highest number of *not very important to me* endorsements: “I learn from agents of change in my community” and “I believe it’s important to live out my faith.” Table 2 shows the scores for each value.

**Table 2:** Student ratings of community-engaged learning (CEL) values: not very important to me (rating=1); neutral (rating=2); and very important to me (rating=3).

Statements	Scores, mean (SD); range
I want to contribute to the good of our society.	2.89 (0.31); 2-3
The skills and knowledge that I gain will help me in my career.	2.85 (0.36); 2-3
I build relationships with people who live and think differently than I do.	2.85 (0.36); 2-3
It makes me a better student in the long run.	2.81 (0.39); 2-3
It helps me build compassion for myself and other people.	2.81 (0.47); 1-3
I can learn more outside the classroom.	2.74 (0.44); 2-3
My assumptions and beliefs are challenged, and I get to challenge others.	2.67 (0.54); 1-3
It empowers me to be an agent of change.	2.63 (0.48); 2-3
I see my community in new ways.	2.56 (0.50); 2-3
It informs the way I engage with the world.	2.56 (0.57); 1-3
I learn from agents of change in my community.	2.52 (0.50); 2-3
I believe it’s important to live out my faith.	2.44 (0.63); 1-3

### Older Adults

#### Pretest-Posttest Analyses

**Figure 3:**
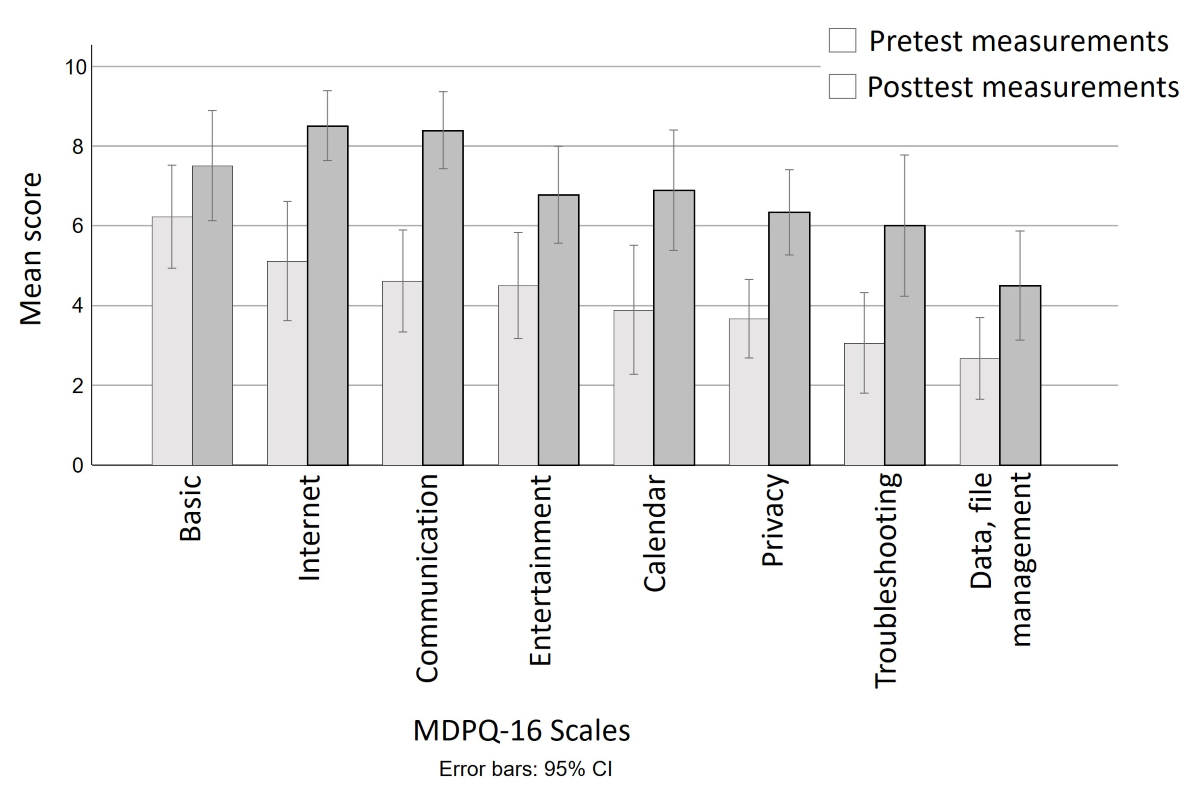
Technology proficiency at pretest and posttest by Mobile Device Proficiency Questionnaire (MDPQ-16) scale.

The means of the summary scores are presented in Table 3. Overall, digital proficiency was analyzed in 2 ways. First, the MDPQ-16 scores were summed across the 16 digital tasks to indicate changes in overall proficiency. We found that scores changed significantly from pretest (mean 33.72, SD 14.05) to posttest measurements (mean 54.89, SD 14.42; t_17_=7.88, *P*<.001). Second, we assessed the changes in the range of activities performed by analyzing scores of 1 (never tried) on the MDPQ-16. Across all 6 activities, the number of “never tried” responses decreased from pretest (mean 9.61, SD 3.60) to posttest measurements (mean 3.83, SD 3.31), representing a significant change on a Wilcoxon signed-rank test of −3.73 (*P*<.001). Finally, we also examined the individual scales on the MDPQ-16 and found that all but basic skills (t_17_=1.48, *P*=.16) increased from pretest to posttest measurements, with *P*<.01 for the remaining 5 scales. Figure 3 shows the scores for all 6 scales at both assessment times.

**Table 3:** Pretest-posttest summary variables for older adults (n=18).

Variables (range of possible scores)	Pretest measurements, mean (SD)	Posttest measurements, mean (SD)	Test statistic^a^	*P* value
MDPQ-16^b^ total (16-80)	33.72 (14.05)	54.89 (14.42)	2.86	<.001
Never tried activities (0-16)	9.61 (3.60)	3.83 (3.31)	−3.73	<.001
Loneliness (3-9)	6.22 (2.58)	7.50 (2.77)	1.41	.18
Attitudes Toward Own Aging (0-5)	2.72 (1.74)	3.44 (1.50)	2.85	.01

^a^All tests are *t* test except “Never tried activities,” which was tested using the Wilcoxon signed-rank test.

^b^MDPQ-16: Mobile Device Proficiency Questionnaire-16.

Loneliness scores were summed across the 3 items (possible scores of 3 to 9), with higher scores indicating greater loneliness. As shown in Table 3, loneliness scores did not change significantly from pretest (mean 6.22, SD 2.58) to posttest measurements (mean 7.50, SD 2.77; t_17_=1.41, *P*=.18). Responses on the 5 Attitudes Toward Own Aging items were summed to create an overall score from 0 to 5, with higher scores reflecting more positive attitudes. We found significant improvements in attitudes toward aging from the pretest (mean 2.72, SD 1.74) to posttest measurements (mean 3.44, SD 1.50; t_17_=2.85, *P*=.01).

#### Posttest Analyses

Relationship quality ratings were positive, with 94% (17/18) indicating that older adults strongly agreed (highest possible score) that they enjoyed working with their student trainer, 89% (16/18) strongly agreed that they formed a close relationship with their student trainer, and similarly 89% (16/18) strongly agreed that they would miss interacting with their student trainer after the project ended (Multimedia Appendix 2). More than three-quarters of older adults (16/18, 89%) responded that they felt “a lot” more socially connected during the technology training program, with the remainder (2/18, 11%) indicating that they felt “a little” more connected. Older adults’ ratings of their computer abilities and confidence were also positive. Almost three-quarters of participants strongly agreed (highest endorsement) that their laptop skills had improved (13/18, 72%), that their confidence in using their laptop had improved (14/18, 78%), and that they were more independent when using their laptop (14/18, 78%). In terms of the overall evaluation of the training program, 100% (18/18) indicated their experience with the training program was very positive (highest endorsement) and 94% (17/18) indicated that the program was very valuable (highest endorsement). Finally, when asked how likely they would be to recommend the training program to others, 94% (17/18) responded extremely likely (highest endorsement) and 6% (1/18) responded very likely.

## Discussion

### Principal Findings

Multifactorial approaches are sorely needed to reduce the growing digital divide among low-income older adults [3,14,18]. The objective of the CEL study presented here was to address 1 component of the digital divide, digital literacy, using a potentially scalable approach. We sought to develop and implement a digital literacy training program using a CEL approach to bring together college students and low-income older adults in the community. There are several notable findings from this work.

First, we found significant improvements in digital literacy as assessed using the MDPQ-16, a measure of computer proficiency that is validated for older adults [57]. To our knowledge, proficiency has not been assessed in past work on training, regardless of whether the program used an intergenerational approach [23,48,49]. In addition to greater proficiency, we also found a significant increase in the breadth of technology use as reflected by a sharp decrease in the number of never-been-tried activities after training. This finding is consistent with intergenerational and nonintergenerational training studies, showing an increased frequency of technology use across multiple tasks [23,49]. However, another study using a similar approach (ie, the item, use of technology in many ways) showed no change following intergenerational training [48]. Overall, our findings add to a small but growing literature showing that intergenerational technology training programs can be an effective approach to improving digital literacy [25] and add to this literature by extending the findings to include digital literacy benefits for low-income older adults.

Second, we found significant improvements in older adults’ confidence surrounding technology use, which is a critical component of technology acceptance and adoption [16]. This finding is consistent with past technology training studies showing improvements in older adults’ confidence [23] and enjoyment of working with technology [48]. Because we included assessments of both confidence and skill, this study extends prior research by showing that both can improve when using an intergenerational approach to promoting digital inclusion.

Third, our results showed beneficial effects on how older adults think about their own aging. Although past work has shown that intergenerational contact can promote positive attitudes among younger individuals [46], we are unaware of prior studies that examined these attitudes among older adults. Past research has shown that older adults’ positive attitudes toward their own aging protect against multiple diseases, including dementia [47,60]. Thus, policy makers interested in tackling the challenges of an aging population should consider investing in intergenerational programs to foster positive attitudes toward aging and enhance older adults’ well-being.

Fourth, findings from this study did not show significant improvements in loneliness from pretest to posttest measurements. Although this was somewhat surprising, other training studies have also shown no effect of training on older adults’ loneliness [23]. Indeed, a review of the literature on the effects of technology interventions, broadly defined, concluded that their impact on older adults’ loneliness is ambiguous [61]. One reason for our findings could be that the pretest survey was administered at the start of the program when older adults had already met their trainers and worked within the same room as other trainers and trainees at the senior center. By contrast, shorter training programs (eg, 8 weeks compared with several months) tend to show null effects on loneliness [61]. Although ours was a group program (in which participants met in a setting with several other individuals), it remains unclear whether loneliness levels would have been impacted by the training program. Therefore, future research is needed to explore the effects of group training programs on loneliness in older adults.

Finally, older adults’ end-of-program ratings suggest that the training program was a success. Self-reported improvements in digital literacy, program value, and program enjoyment were all high, and 94% (17/18) of older adults indicated that they were extremely likely to recommend the program to others. Enthusiasm for working with college student trainers has been reported in the past work [25,48]. High satisfaction with our digital literacy training programs may also be because the low-income older adults in our study (1) were able to keep the laptop and (2) received funds for a 2-year broadband subscription following the training (this support was for a federal low-income program called the Affordable Connectivity Program). This may also have helped to increase engagement and commitment to learning how to use the technology. Policy makers and community organizations interested in bridging the digital divide among low-income older populations should provide tangible support, such as digital devices and broadband connectivity to enhance program outcomes and promote continued digital engagement.

The significant improvements observed in older adults’ digital proficiency, confidence in technology use, and attitudes toward aging underscore the potential of intergenerational approaches that bring together older adults and college students to promote digital inclusion and well-being among older adults. Programs that help to formalize opportunities for undergraduates to work with low-income older adults as part of their undergraduate education (rather than in addition to it) may be particularly impactful for both older and younger adults. In subsequent sections, we outline some of these benefits.

### CEL Approach

#### Reduces Uncertainties Surrounding Logistics of Meeting Times and Place

The prescheduled meeting times and a meeting place at a local senior center provided structure, which reduced uncertainties for both students and older adults. Data from our preliminary study indicated that both students and older adults were frustrated and confused by scheduling constraints and last-minute changes.

#### Train the Trainer

Another advantage to this approach is the ability to focus on training the trainers, which in this study included students learning about design thinking and aging. The ongoing educational support to enable students to learn how to train older adults may be even more important when working with low-income older adults. Although other technology training programs have included educational support for students, they included only 1 session [48] or 1 session along with an optional social media forum for student trainers to support each other [49]. In this study, students’ questions and observations about how to tailor the training to be more effective continued throughout the duration of the program.

#### Focus on Social Good

As with public psychology [62], CEL is concerned with social good and the welfare of others. This importance of social good is becoming increasingly acknowledged in other public and private institutions of higher education [63]. In this study, we found that the students were also concerned with social good. CEL ratings reflect students’ interest in community, social welfare, and serving those in need. Developmentally, young adults tend to be concerned with the need for social change and identification of ways in which they can make a difference [51]. CEL with an intergenerational focus provides an opportunity for younger adults to express their concerns and broaden their awareness of social needs via exposure to underserved older adults. Furthermore, when CEL is embedded within the curriculum, students can more easily take advantage of opportunities to “give back” while also working toward their academic goals. This may be particularly important for underrepresented students who are often unable to do internships for a variety of reasons, including strict timelines for graduation, work obligations (sometimes several jobs), and family responsibilities.

#### Community and Campus Relationships

CEL relies heavily on close working ties with community members. In this study, the most important community members were the low-income older adults. Indeed, the relationship between the older adult trainees and the student trainers played a critical role in the success of the program. Community organizations also play a critical role and can help with recruiting older adults; donating space and associated services (eg, tables, chairs, parking, signage, and communication); and applying for funding to purchase laptops and broadband subscriptions. Finally, campus stakeholders also play a central role in the developing, testing, and scaling up of programs that focus on underserved older adults in the community. For example, campus leaders can express their enthusiasm for CEL courses, provide CEL experts to support instructors interested in designing courses, develop relationships with community stakeholders, and provide financial incentives (eg, to programs and departments) to incorporate these courses into existing degree requirements. The importance of campus and community partnerships cannot be underestimated [64]. Future work should therefore consider innovative ways to engage community organizations and campus leaders in efforts to build effective and sustainable intergenerational programs that improve low-income older adults’ digital literacy.

### Intergenerational Approach

This study used an intergenerational approach to serving low-income older adults. As suggested by the posttest scores, students valued getting to know their older adult trainees. Their experiences across the program led to greater confidence in working with older adults, as has been shown in past work [48]. In addition, consistent with past research, we did not find significant changes in fear of older adults (*P*=.42) [44,48]. Surprisingly, however, we did not find significant improvement in students’ attitudes toward aging as has been reported in the past assessments [48]. A closer look at the prior study indicates that students in this study scored as high on the Psychological Growth scale at the pretest time point (mean 29.58, SD 3.11) as those in the study by Leedahl et al [48] at the posttest time point (mean 29.42, SD 3.19), suggesting that there was little room for improvement as a result of their interactions across the training program. In addition to whether changes in attitudes occur within an academic term, it is important to consider the possibility of longer-term effects of intergenerational programs, including CEL. In this study, the first-year seminar course had no prerequisites, potentially attracting those who would not otherwise consider working with older adults. Given an increasingly age-segregated society [40] and a severe shortage of individuals trained to work in fields related to aging [65], this approach may promote interest among students to work on solving some of the pressing issues in facing a world with an unprecedented number of older adults [66,67].

### Limitations

There were several limitations of our study. First, because of our single-group design, we cannot know which components of the training program were responsible for the beneficial outcomes. For example, we cannot disentangle the effects of training from the effects of owning a new laptop or assume the intergenerational component is superior to other models of training (eg, the use of technology experts). Second, our pretest measures might have shown a stronger impact on the program had we administered them 1 or 2 weeks before the start of the training program. Because we assessed them in the first 2 weeks, it is possible that some outcomes, such as loneliness and basic technology skills, might have already improved. Third, as with some previous training studies [25,48], the sample size was small, which can limit the ability to detect smaller effects. The sample was also predominantly consisted of White and female individuals, limiting the generalizability of the findings. Our sample does not, for example, allow us to account for the potential role that sociocultural factors (eg, culture, country of origin, intersecting identities, situations, or the interplay between these factors) play in the augmentation of digital literacy in older adults. Importantly, however, participants in this study were from low-income households, which are significantly underrepresented in the literature. Finally, our study did not include a follow-up to examine the long-term impact of training on new technology adoption and sustained use of the laptops. It is also possible, for example, that greater digital literacy skills would lead to decreased loneliness over time as older adults begin to use technology for social interactions [68]. Additional work is needed to explore the longer-term impact and scalability of this approach to promoting digital literacy among low-income older adults and to examine other potential benefits to both older and younger adults.

### Conclusions

Taken together, the current research contributes to a growing body of research on digital literacy training and provides a potential pathway to address the digital divide among underserved older adults [3,14,18]. Digital inclusion is central to older adults’ ability to remain independent and live in their own homes as they age. Moreover, the intergenerational CEL approach used in this study promotes mutual respect across generations, breaks down harmful stereotypes, and helps to build a stronger community. Moving forward, continued research in this area is crucial for informing policy decisions that support digital inclusion for older adults and help to address broader challenges related to an aging global population, digital fairness, social justice, and the shared fate of humanity.

## Appendix

Multimedia Appendix 1 Digital literacy training content and process overview that students used while training older adults.

Multimedia Appendix 2 Older adults’ posttest-only data.

## Abbreviations

CEL: community-engaged learning
CREATE: Center for Research and Education on Aging and Technology Enhancement
MDPQ-16: Mobile Device Proficiency Questionnaire
STAM: Senior Technology Acceptance and Adoption Model

## References

[1] Philbin MM, Parish C, Pereyra M, Feaster DJ, Cohen M, Wingood G, Konkle-Parker D, Adedimeji A, Wilson TE, Cohen J, Goparaju L, Adimora AA, Golub ET, Metsch LR (2019). Health disparities and the digital divide: the relationship between communication inequalities and quality of life among women in a nationwide prospective cohort study in the United States. J Health Commun.

[2] Sieck CJ, Sheon A, Ancker JS, Castek J, Callahan B, Siefer A (2021). Digital inclusion as a social determinant of health. NPJ Digit Med.

[3] Singh GK, Girmay M, Allender M, Christine RT (2020). Digital divide: marked disparities in computer and broadband internet use and associated health inequalities in the United States. Int J Transl Med Res Public Health.

[4] Drazich BF, Lee JW, Bowles KH, Taylor JL, Shah S, Resnick B, Kim N, Szanton SL (2023). Pandemic-related changes in technology use among a sample of previously hospitalized older adult New Yorkers: observational study. JMIR Aging.

[5] Gauthier GR, Smith JA, García C, Garcia MA, Thomas PA (2021). Exacerbating inequalities: social networks, racial/ethnic disparities, and the COVID-19 pandemic in the United States. J Gerontol B Psychol Sci Soc Sci.

[6] Jaffe DH, Lee L, Huynh S, Haskell TP (2020). Health inequalities in the use of telehealth in the United States in the lens of COVID-19. Popul Health Manag.

[7] Seifert A, Cotten SR, Xie B (2021). A double burden of exclusion? Digital and social exclusion of older adults in times of COVID-19. J Gerontol B Psychol Sci Soc Sci.

[8] Molinsky J, Herbert C, Forsyth A, Coll P (2019). Housing and planning supporting healthy aging. Healthy Aging.

[9] National Academies of Sciences, Engineering, and Medicine, Division of Behavioral and Social Sciences and Education, Health and Medicine Division, Board on Behavioral, Cognitive, and Sensory Sciences, Board on Health Sciences Policy (2020). Social Isolation and Loneliness in Older Adults: Opportunities for the Health Care System.

[10] Daly JR, Depp C, Graham SA, Jeste DV, Kim HC, Lee EE, Nebeker C (2021). Health impacts of the stay-at-home order on community-dwelling older adults and how technologies may help: focus group study. JMIR Aging.

[11] Choi NG, Dinitto DM (2013). The digital divide among low-income homebound older adults: internet use patterns, eHealth literacy, and attitudes toward computer/internet use. J Med Internet Res.

[12] Gordon NP, Crouch E (2019). Digital information technology use and patient preferences for internet-based health education modalities: cross-sectional survey study of middle-aged and older adults with chronic health conditions. JMIR Aging.

[13] Jensen JD, King AJ, Davis LA, Guntzviller LM (2010). Utilization of internet technology by low-income adults: the role of health literacy, health numeracy, and computer assistance. J Aging Health.

[14] Marston HR, Shore L, White PJ (2020). How does a (smart) age-friendly ecosystem look in a post-pandemic society?. Int J Environ Res Public Health.

[15] Mitchell UA, Chebli PG, Ruggiero L, Muramatsu N (2019). The digital divide in health-related technology use: the significance of race/ethnicity. Gerontologist.

[16] Czaja SJ, Charness N, Fisk AD, Hertzog C, Nair SN, Rogers WA, Sharit J (2006). Factors predicting the use of technology: findings from the center for research and education on aging and technology enhancement (CREATE). Psychol Aging.

[17] Czaja SJ, Boot WR, Charness N, Rogers WA, Sharit J (2018). Improving social support for older adults through technology: findings from the PRISM randomized controlled trial. Gerontologist.

[18] Ellison-Barnes A, Moran A, Linton S, Chaubal M, Missler M, Evan Pollack C (2021). Limited technology access among residents of affordable senior housing during the COVID-19 pandemic. J Appl Gerontol.

[19] Golomski C, Corvini M, Kim B, Wilcox J, Valcourt S (2022). Aspects of ICT connectivity among older adults living in rural subsidized housing: reassessing the digital divide. J Enabling Technol.

[20] Kebede AS, Ozolins LL, Holst H, Galvin K (2022). Digital engagement of older adults: scoping review. J Med Internet Res.

[21] López Seguí F, de San Pedro M, Aumatell Verges E, Simó Algado S, Garcia Cuyàs F (2019). An intergenerational information and communications technology learning project to improve digital skills: user satisfaction evaluation. JMIR Aging.

[22] Wu YH, Damnée S, Kerhervé H, Ware C, Rigaud AS (2015). Bridging the digital divide in older adults: a study from an initiative to inform older adults about new technologies. Clin Interv Aging.

[23] Fields J, Cemballi AG, Michalec C, Uchida D, Griffiths K, Cardes H, Cuellar J, Chodos AH, Lyles CR (2021). In-home technology training among socially isolated older adults: findings from the tech allies program. J Appl Gerontol.

[24] Cotten SR (2021). Technologies and aging: understanding use, impacts, and future needs. Handbook of Aging and the Social Sciences (Ninth Edition).

[25] Arthanat S, Vroman KG, Lysack C (2016). A home-based individualized information communication technology training program for older adults: a demonstration of effectiveness and value. Disabil Rehabil Assist Technol.

[26] Campbell RJ, Nolfi DA (2005). Teaching elderly adults to use the internet to access health care information: before-after study. J Med Internet Res.

[27] Chan MY, Haber S, Drew LM, Park DC (2016). Training older adults to use tablet computers: does it enhance cognitive function?. Gerontologist.

[28] Quialheiro A, Miranda A, Garcia M Jr, Carvalho AC, Costa P, Correia-Neves M, Santos NC (2023). Promoting digital proficiency and health literacy in middle-aged and older adults through mobile devices with the workshops for online technological inclusion (OITO) project: experimental study. JMIR Form Res.

[29] Tsai HY, Rikard RV, Cotten SR, Shillair R (2019). Senior technology exploration, learning, and acceptance (STELA) model: from exploration to use – a longitudinal randomized controlled trial. Educ Gerontol.

[30] Charness N, Boot WR (2022). A grand challenge for psychology: reducing the age-related digital divide. Curr Dir Psychol Sci.

[31] Renaud K, van Biljon J (2008). Predicting technology acceptance and adoption by the elderly: a qualitative study. Proceedings of the 2008 annual research conference of the South African Institute of Computer Scientists and Information Technologists on IT research in developing countries: riding the wave of technology.

[32] Czaja SJ, Boot WR, Charness N, Rogers WA (2019). Designing for Older Adults: Principles and Creative Human Factors Approaches, Third Edition.

[33] Jung Y, Peng W, Moran M, Jin SA, McLaughlin M, Cody M, Jordan-Marsh M, Albright J, Silverstein M (2010). Low-income minority seniors' enrollment in a cybercafé: psychological barriers to crossing the digital divide. Educ Gerontol.

[34] Klimova B (2016). Computer-based cognitive training in aging. Front Aging Neurosci.

[35] Tappen RM, Cooley ME, Luckmann R, Panday S (2022). Digital health information disparities in older adults: a mixed methods study. J Racial Ethn Health Disparities.

[36] Bandura A (1989). Regulation of cognitive processes through perceived self-efficacy. Dev Psychol.

[37] Miller LM, Lachman ME (1999). The sense of controlcognitive aging: toward a model of mediational processes. Social Cognition and Aging.

[38] Gruenewald TL, Tanner EK, Fried LP, Carlson MC, Xue QL, Parisi JM, Rebok GW, Yarnell LM, Seeman TE (2016). The Baltimore experience corps trial: enhancing generativity via intergenerational activity engagement in later life. J Gerontol B Psychol Sci Soc Sci.

[39] Murayama Y, Ohba H, Yasunaga M, Nonaka K, Takeuchi R, Nishi M, Sakuma N, Uchida H, Shinkai S, Fujiwara Y (2015). The effect of intergenerational programs on the mental health of elderly adults. Aging Ment Health.

[40] Riley MW, Riley JW Jr (2000). Age integration: conceptual and historical background. Gerontologist.

[41] Wilmoth JM, Longino CF Jr (2006). Demographic trends that will shape U.S. Policy in the twenty-first century. Res Aging.

[42] Martins T, Midão L, Martínez Veiga S, Dequech L, Busse G, Bertram M, McDonald A, Gilliland G, Orte C, Vives M, Costa E (2018). Intergenerational programs review: study design and characteristics of intervention, outcomes, and effectiveness. J Intergenerational Relatsh.

[43] Canedo-García A, García-Sánchez JN, Pacheco-Sanz DI (2017). A systematic review of the effectiveness of intergenerational programs. Front Psychol.

[44] Andreoletti C, Howard JL (2018). Bridging the generation gap: intergenerational service-learning benefits young and old. Gerontol Geriatr Educ.

[45] Breck BM, Dennis CB, Leedahl SN (2018). Implementing reverse mentoring to address social isolation among older adults. J Gerontol Soc Work.

[46] Burnes D, Sheppard C, Henderson CR Jr, Wassel M, Cope R, Barber C, Pillemer K (2019). Interventions to reduce ageism against older adults: a systematic review and meta-analysis. Am J Public Health.

[47] Levy BR, Slade MD, Chang ES, Kannoth S, Wang SY (2020). Ageism amplifies cost and prevalence of health conditions. Gerontologist.

[48] Leedahl SN, Brasher MS, Estus E, Breck BM, Dennis CB, Clark SC (2018). Implementing an interdisciplinary intergenerational program using the Cyber Seniors reverse mentoring model within higher education. Gerontol Geriatr Educ.

[49] Arthanat S (2021). Promoting information communication technology adoption and acceptance for aging-in-place: a randomized controlled trial. J Appl Gerontol.

[50] Mitchell T (2008). Traditional vs. critical service-learning: engaging the literature to differentiate two models. Mich J Community Serv Learn.

[51] Institute of Medicine and National Research Council (2015). Investing in the Health and Well-Being of Young Adults.

[52] Adams C, Nash JB (2016). Exploring design thinking practices in evaluation. J Multidiscip Eval.

[53] Panke S (2019). Design thinking in education: perspectives, opportunities and challenges. Open Educ Stud.

[54] Lubben J, Blozik E, Gillmann G, Iliffe S, von Renteln Kruse W, Beck JC, Stuck AE (2006). Performance of an abbreviated version of the Lubben social network scale among three European community-dwelling older adult populations. Gerontologist.

[55] Laidlaw K, Kishita N, Shenkin SD, Power MJ (2018). Development of a short form of the attitudes to ageing questionnaire (AAQ). Int J Geriatr Psychiatry.

[56] Lasher KP, Faulkender PJ (1993). Measurement of aging anxiety: development of the anxiety about aging scale. Int J Aging Hum Dev.

[57] Roque NA, Boot WR (2018). A new tool for assessing mobile device proficiency in older adults: the mobile device proficiency questionnaire. J Appl Gerontol.

[58] Hughes ME, Waite LJ, Hawkley LC, Cacioppo JT (2004). A short scale for measuring loneliness in large surveys: results from two population-based studies. Res Aging.

[59] Lawton MP (1975). The Philadelphia geriatric center morale scale: a revision. J Gerontol.

[60] Levy BR, Slade MD, Pietrzak RH, Ferrucci L (2018). Positive age beliefs protect against dementia even among elders with high-risk gene. PLoS One.

[61] Balki E, Hayes N, Holland C (2022). Effectiveness of technology interventions in addressing social isolation, connectedness, and loneliness in older adults: systematic umbrella review. JMIR Aging.

[62] Eaton AA, Grzanka PR, Schlehofer MM, Silka L (2021). Public psychology: introduction to the special issue. Am Psychol.

[63] Jacob WJ, Sutin S, Weidman JL, Yeager JC (2015). Community Engagement in Higher Education: Policy Reforms and Practice.

[64] Roodin P, Brown LH, Shedlock D (2013). Intergenerational service-learning: a review of recent literature and directions for the future. Gerontol Geriatr Educ.

[65] Rowe JW (2021). The US eldercare workforce is falling further behind. Nat Aging.

[66] Christensen K, Doblhammer G, Rau R, Vaupel JW (2009). Ageing populations: the challenges ahead. Lancet.

[67] Gu D, Andreev K, Dupre ME (2021). Major trends in population growth around the world. China CDC Wkly.

[68] Aarts S, Peek ST, Wouters EJ (2015). The relation between social network site usage and loneliness and mental health in community-dwelling older adults. Int J Geriatr Psychiatry.

