# Supplementary material for: Digital Literacy Training for Low-Income Older Adults Through Undergraduate Community-Engaged Learning: Single-Group Pretest-Posttest Study
Source: JMIR Aging. 2024 May 14;7:e51675. doi: 10.2196/51675 (PMC11134247; doi:10.2196/51675)

**Appendix A. Digital Literacy Training Content and Process Overview that Students Used While Training Older Adults**

**Training Content**

**Basic Tasks** (These are suggestions only; listen to trainee’s interests and goals to identify additional tasks)

1. Getting to know the laptop (hardware, touch screen, typing, voice dictation)
2. Email (sending email, using the camera, making screenshots)
3. Using the laptop (getting online, searching for information)
4. Online safety (passwords, phishing, viruses)
5. Email safety (opening and replying to emails, identifying spam)
6. Optional:

- Communicating via video phone apps
- Online communities (social media; caregiving support)
- Other topics may also work; check with me to discuss

1. During **last two weeks** of training, show trainee how to sign up for and use the *Area 4 on Aging* digital learning site; select a few courses in advance to add to their Learner Booklet; demonstrate how to join an “in progress” course and show them how to ask questions.

**How to Create Practice Activities**

- Practice activities should be centered on what you’ve recently taught them in one of your sessions, rather than something that they may not yet know well enough to practice on their own.
- Tasks should be well-defined and simple (which is relative to your trainee’s ability) rather than vague and challenging.
- Tasks should have a verifiable result so you and the trainee know whether the task was done correctly.
- The number times they complete a task could vary (possibly 2-4 times/wk), depending on the task and how difficult it is for the trainee to perform.
- Encourage their practice to be spread out across the week (distributed) rather than crammed into one or two days.

*Example:*

Sending screenshot of something on their laptop (e.g., an error message that appeared, something they have a question about/need help with). STEPS: Take a screen shot, find where the image is saved, open email app, create new email message, attach image to email, and send. Could ask them to practice this twice before the next session.

**Training Process**

In each **training session**, greet your trainee by name (ask them for preferred title (Mr/Ms) or name) and check in with them (how are they doing, etc). Then use the following outline to guide training: start with the past week, move on to the present, and then discuss what comes next (future) as described here:

**Past**

How did the practice go last week? **Verify learning:** have them show you what they did during practice last week so you can see 1) if it was done correctly and 2) where the hard parts are, if any. If they had difficulty, review the steps that you created last time and make adjustments (add reminders) to help them learn. (Revised steps will go into the final project). Even if you do not see difficulty, ask for their **feedback** on the instructions you provided (e.g., were they clear, detailed enough, too much detail?)

**Present**

Decide if your time is better spent on a prior task or move on to a new one. Circle back to an earlier task to show overlap with each new task. Use the **MDPQ-16** in the first few sessions to spur discussion which will enable you to understand a) trainee’s current skills and b) the context in which they use these skills (e.g., who, what, where, when, and why). Understanding this context enables you to more specifically tailor the training to this individual. Refer back to this information and the **Basic Tasks** list above for training ideas across the quarter. To prepare for our class discussions, use training time to think about design challenges you observe (e.g., devices, apps, training itself) and the extent to which what they do/how they do it could be improved to better meet your trainee’s needs.

**To begin teaching a new activity, try showing them on their laptop or walk them through by providing verbal step-by-step instructions**. Write down the steps that are needed to perform the task successfully and add images (i.e., screen shots with arrows pointing at where to look) to facilitate learning. Type the instructions into an email (and review them with trainee for clarity) then send the email. This way, you both have a record. (It is important for you to have a record because these instructions are needed for your Post-Session Record entry, which will form the basis of the Learner Booklet (final project)). As you teach, **verify learning**: have trainee perform the task, watch for errors (or less-than-ideal ways of doing a task), and identify ways to minimize/avoid these. If they are learning quickly, still provide some practice, but also move on to the next training task. If they are struggling, break down the task into smaller pieces and practice each with them over and over again, gradually providing less support (fewer hints) as you go along. Keep track of what seemed to help them most (what or how you communicated) so you can incorporate these ideas into their Learner Booklet.

**Future**

Save at least 10 minutes at the end of the session to review the topic(s) covered today and, working together, define the steps used to complete tasks for the topic(s). Discuss where to pick up next time with the trainee (e.g., a new topic vs more practice or greater depth of current topic). Type up a **summary** of the session in an email to the trainee and **plans** for next session. (Note, you can always change plans when at next session; for now, make your best guess of what you and your trainee should work on next).

**Appendix B. Older Adults’ Post-test Only Data**


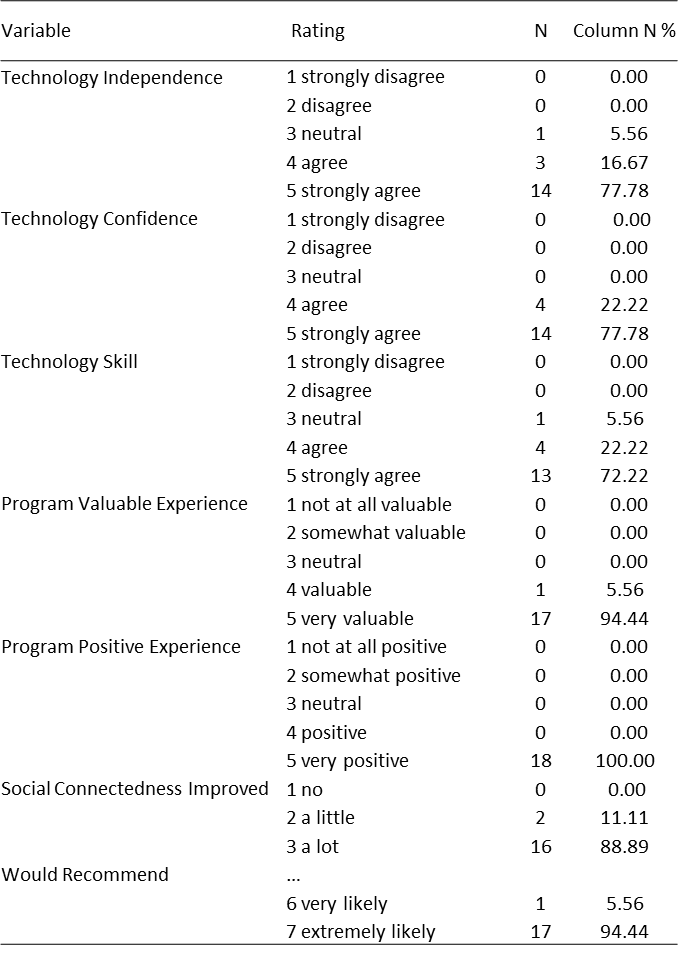

Supplement: Multimedia Appendix 1 [file aging_v7i1e51675_app1.docx]
