# Supplementary figures and images for: Digital Literacy Training for Low-Income Older Adults Through Undergraduate Community-Engaged Learning: Single-Group Pretest-Posttest Study
Source: JMIR Aging. 2024 May 14;7:e51675. doi: 10.2196/51675 (PMC11134247; doi:10.2196/51675)

**Appendix B. Older Adults’ Post-test Only Data**


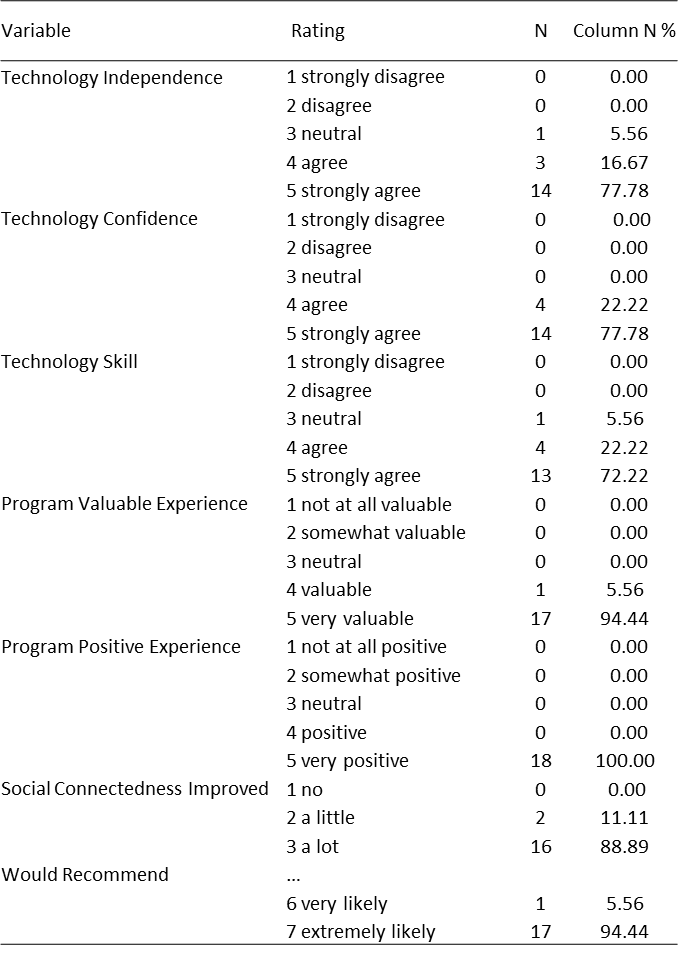

Supplement: Multimedia Appendix 2 [file aging_v7i1e51675_app2.docx]
